# Highly Efficient Electrocatalyst of 2D–2D gC_3_N_4_–MoS_2_ Composites for Enhanced Overall Water Electrolysis

**DOI:** 10.3390/ma18163775

**Published:** 2025-08-12

**Authors:** Sankar Sekar, Atsaya Shanmugam, Youngmin Lee, Sejoon Lee

**Affiliations:** 1 Division of System Semiconductor, Dongguk University, Seoul 04620, Republic of Korea; sanssekar@dongguk.edu (S.S.); atsyshanmu@dgu.ac.kr (A.S.); 2 Quantum-Functional Semiconductor Research Center, Dongguk University, Seoul 04620, Republic of Korea

**Keywords:** molybdenum disulfide, graphitic carbon nitride, nanocomposites, electrocatalysts, bifunctional water electrolysis

## Abstract

For future clean and renewable energy technology, designing highly efficient and robust electrocatalysts is of great importance. Particularly, creating efficient bifunctional electrocatalysts capable of effectively catalyzing both hydrogen- and oxygen-evolution reactions (HERs and OERs) is vital for overall water electrolysis. In this study, we employ 2D molybdenum disulfide (MoS_2_) nanosheets and pyrolytically fabricated 2D graphitic carbon nitride (gC_3_N_4_) nanosheets to create 2D gC_3_N_4_-decorated 2D MoS_2_ (2D–2D gC_3_N_4_–MoS_2_) nanocomposites using a facile sonochemical method. The 2D–2D gC_3_N_4_–MoS_2_ nanocomposites show an interconnected and agglomerated structure of 2D gC_3_N_4_ nanosheets decorated on 2D MoS_2_ nanosheets. For water electrolysis, the gC_3_N_4_–MoS_2_ nanocomposites exhibit low overpotentials (OER: 225 mV, HER: 156 mV), small Tafel slope values (OER: 49 mV/dec, HER: 101 mV/dec), and excellent durability (up to 100 h for both OER and HER) at 10 mA/cm^2^ in 1 M KOH. Furthermore, the gC_3_N_4_–MoS_2_ nanocomposites show excellent overall water electrolysis performance with a low full-cell voltage (1.52 V at 10 mA/cm^2^) and outstanding long-term cell stability. The superb bifunctional activities of the gC_3_N_4_–MoS_2_ nanocomposites are attributed to the synergistic effects of 2D gC_3_N_4_ (i.e., low charge-transfer resistance) and 2D MoS_2_ (i.e., a large electrochemically active surface area). These findings suggest that the 2D–2D gC_3_N_4_–MoS_2_ nanocomposites could serve as excellent bifunctional catalysts for overall water electrolysis.

## 1. Introduction

Fossil fuel scarcity, increasing energy demands, and environmental pollution concerns have stimulated significant interest in pursuing renewable, eco-friendly, and clean energy sources [1,2,3,4]. Recently, hydrogen has emerged as a leading alternative renewable energy resource because of its excellent mass–energy density, pollution-free characteristics, sustainability, renewability, and superior cleanliness [5,6,7,8]. Among all the available hydrogen production methods, electrocatalytic water electrolysis is considered a fascinating method for harvesting oxygen and hydrogen energies from water (via oxygen- and hydrogen-evolution reactions (OERs and HERs), respectively) because of its environmental friendliness, reusability, scalability, and feasibility [9,10,11,12]. Currently, Pt (HER catalyst) and IrO_2_/RuO_2_-based (OER catalyst) materials are widely believed to be highly efficient, stable, and reliable catalysts for water splitting, but their material scarcity and high cost hinder their practical applications [13,14]. Most of the reported electrocatalysts have not yet exhibited outstanding OER and HER activities simultaneously in a single electrolyte because of incompatible activity and stability [15,16,17,18]. Therefore, developing alternative catalysts with superior durability, excellent catalytic active sites, low cost, and earth-abundant resources is vital.

Recently, transition metal dichalcogenides (TMDs)-based materials have garnered considerable attention as prospective electrocatalysts for OERs and HERs because of their distinctive two-dimensional (2D) layered structure, low cost, and unique physicochemical characteristics [19,20,21,22]. Among the various TMDs, molybdenum disulfide (MoS_2_) is considered a substantial catalyst for water splitting owing to its high intrinsic edge activity, layered structure, excellent stability, nontoxicity, natural abundance, low cost, unique electronic structures, and good electrochemical catalytic activity [23,24,25]. Generally, MoS_2_ comprises two phases, i.e., 2H (semiconducting) and 1T (metallic) [26]. However, pure 2H phase MoS_2_ demonstrates insufficient electrocatalytic performance due to its inferior electrical conductivity, insufficient active edge sites, sluggish charge-transfer kinetics, and high hydrogen adsorption–desorption energy [27,28]. Therefore, various methods have been used to hybridize 2H MoS_2_ nanostructures with transition metal/metal oxide-based materials [29,30,31,32,33] and carbonaceous materials [34,35,36,37,38] in order to improve their electrochemical catalytic behavior. Recently, graphitic carbon nitride (gC_3_N_4_) has gained significant interest as a good co-catalyst for enhancing the catalytic activity of host catalysts owing to its impressive chemical stability, facile synthesis, affordability, high earth abundance, unique 2D layered structure, high nitrogen concentration, and easily adaptable structure [39,40]. The coupling of 2D MoS_2_ and 2D gC_3_N_4_ can notably enhance the specific surface area, electrical conductivity, and density of active catalytic sites, all of which are beneficial for improving electrocatalytic performance [34,41]. For example, Fageria et al. [34] hydrothermally synthesized the highly efficient electrocatalyst of MoS_2_-decorated gC_3_N_4_, demonstrating an HER overpotential of 240 mV at −10 mA/cm^2^ in 0.5 M H_2_SO_4_. Additionally, Zhang et al. [35] synthesized MoS_2_ nanosheets anchored on a gC_3_N_4_ substrate, exhibiting an HER overpotential of 158 mV at −10 mA/cm^2^ in 0.5 M H_2_SO_4_. Recently, Liu et al. [42] used the hydrothermal method, followed by ultrasonic techniques, to fabricate a hybrid structure of the MoS_2_/gC_3_N_4_ nanojunction, which exhibited effective HER activity with an overpotential of −200 mV to achieve a current density of 10 mA/cm^2^ in 0.5 M H_2_SO_4_. He et al. [43] fabricated a CoO_x_/mC@MoS_2_@gC_3_N_4_ composite that showed the HER overpotential of 31 mV at −10 mA/cm^2^ in 0.5 M H_2_SO_4_. Furthermore, Mehtab et al. [44] synthesized the MoS_2_/gC_3_N_4_ heterostructure through a ball-milling technique and exhibited bifunctional OER and HER activities with overpotential values of 410 and 262 mV at 20 mA/cm^2^ in 0.1 M KOH, respectively. Despite all the benefits of 2D MoS_2_ and 2D gC_3_N_4_, hybrid 2D gC_3_N_4_-decorated 2D MoS_2_ (2D–2D gC_3_N_4_–MoS_2_) nanocomposites have rarely been investigated for bifunctional water electrolysis, particularly in regards to substantial OER and HER performance in alkaline medium.

Motivated by the abovementioned backgrounds, we synthesized 2D–2D gC_3_N_4_–MoS_2_ nanocomposites using a facile sonochemical method and examined their bifunctional water electrolysis performances. The gC_3_N_4_–MoS_2_ nanocomposites demonstrated excellent electrocatalytic water-splitting activities in 1 M KOH with small overpotential values (i.e., 225 mV for OER and 156 mV for HER at 10 mA/cm^2^). Furthermore, the assembled gC_3_N_4_–MoS_2_||gC_3_N_4_–MoS_2_ electrolyzer demonstrated a low full cell voltage of 1.52 V at a current density of 10 mA/cm^2^, maintaining superior stability during prolonged operation for up to 100 h. Herein, the synthesis-to-electrocatalytic characteristics of the 2D–2D gC_3_N_4_–MoS_2_ nanocomposites are assessed and deliberated in detail.

## 2. Experiment

### 2.1. MoS_2_ Nanosheet Synthesis

**Figure 1:**
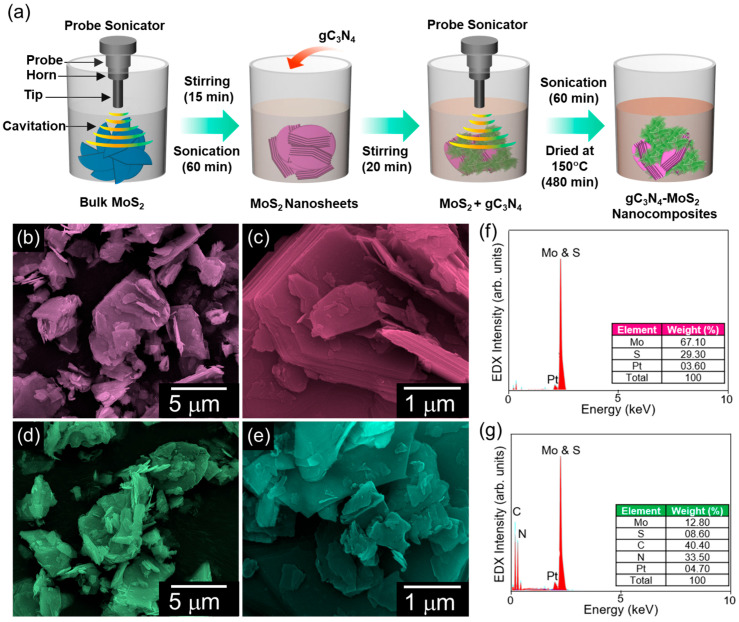
(**a**) Sonochemical fabrication process of the MoS_2_ nanosheets and gC_3_N_4_–MoS_2_ nanocomposites, (**b**,**c**) FE-SEM images of MoS_2_, (**d**,**e**) FE-SEM images of gC_3_N_4_–MoS_2_, and EDX spectra of (**f**) MoS_2_ and (**g**) gC_3_N_4_–MoS_2_.

Figure 1a depicts the facile sonochemical process for producing the hybrid 2D–2D gC_3_N_4_–MoS_2_ nanocomposites. The 2D MoS_2_ nanosheets were derived from commercial bulk MoS_2_ (Sigma-Aldrich, St. Louis, MO, USA). Initially, 2 g of bulk MoS_2_ was dispersed into 100 mL of deionized water and stirred continuously for 30 min. Next, the blended solution was sonicated (*f_ultra_* = 35 kHz and *P_ultra_* = 240 W) for 60 min. During the ultrasonication process, the ultrasonic waves induced alternating low and high pressures in the bulk MoS_2_. This process facilitated cavitation-driven exfoliation of the MoS_2_ sub-lattices, resulting in the successful formation of layered MoS_2_ nanosheets. Afterward, the colloidal suspension was collected, cleaned, filtered, and parched in a 150 °C electric oven for 480 min. Finally, the MoS_2_ nanosheets were collected in powder form.

### 2.2. gC_3_N_4_ Nanosheet Synthesis

The 2D gC_3_N_4_ nanosheets were synthesized from melamine using a facile pyrolysis method [39,45,46]. Initially, 5 g of melamine (Sigma-Aldrich, St. Louis, MO, USA) was transferred to an alumina crucible and encapsulated by the crucible cover. Thereafter, the crucible setup was loaded in a muffle furnace and thermally annealed at 550 °C for 240 min in an air atmosphere. Finally, yellow-colored powdered gC_3_N_4_ nanosheets were obtained.

### 2.3. gC_3_N_4_–MoS_2_ Nanocomposite Fabrication

The 2D–2D gC_3_N_4_–MoS_2_ nanocomposites were fabricated via an ultrasonication process using the pyrolytically fabricated 2D gC_3_N_4_ nanosheets and the sonochemically derived 2D MoS_2_ nanosheets. Prior to discussing the experimental procedures, it is important to note that the gC_3_N_4_ to MoS_2_ ratio of 1:0.3 was selected based on insights from previous literature [47,48,49,50,51,52], as this composition was found to provide optimal material properties and enhanced electrochemical performance. For this, first, the MoS_2_ nanosheets (1 g) were interspersed in deionized water (100 mL) through vigorous stirring for 20 min. Then, the gC_3_N_4_ nanosheets (0.3 g) were subsequently added and mixed with the abovementioned MoS_2_ solution through additional stirring for 20 min. Thereafter, the gC_3_N_4_–MoS_2_ blended aqueous solution was ultrasonicated for 60 min (*f_ultra_* = 35 kHz and *P_ultra_* = 240 W). Finally, the ultrasonicated colloidal suspension was washed, accumulated, and parched at 150 °C for 6 h to obtain the nanopowder form of 2D–2D gC_3_N_4_–MoS_2_.

### 2.4. Material Characterization

The morphological and elemental properties of MoS_2_ and gC_3_N_4_–MoS_2_ were monitored through field-emission scanning electron microscopy (FE-SEM, Clara LMH, Tescan Brno, Czech Republic) and in situ energy-dispersive X-ray spectroscopy (EDX) measurements, respectively. Moreover, the topographic features of the prepared catalysts were further assessed via transmission electron microscopy (TEM, JEM 2100F, JEOL, Tokyo, Japan). Crystallographic information was investigated through the X-ray diffraction (XRD; D8-Advance, Bruker, Billerica, MA, USA) analysis. The textural characteristics were assessed via the Brunauer–Emmett–Teller (BET, BELSORP-mini II system, MicrotracBEL, Osaka, Japan) and Barrett–Joyner–Halenda (BJH) techniques. The surface chemical states of the synthesized catalysts were examined through X-ray photoelectron spectroscopy (XPS, ESCALab250Xi system, Thermos Fisher Scientific, Waltham, MA, USA).

### 2.5. Electrocatalytic Measurements

The water splitting performance of the prepared MoS_2_ nanosheets and gC_3_N_4_–MoS_2_ nanocomposites was evaluated using the typical three-electrode method in an alkaline electrolyte (1 M KOH) solution using the VersaSTAT3 workstation (Ametek Scientific Company, Mahwah, NJ, USA). First, we assembled two different working electrodes using the prepared 2D MoS_2_ nanosheets and 2D–2D gC_3_N_4_–MoS_2_ nanocomposites. For this, 3 mg of each synthesized catalyst (i.e., either MoS_2_ or gC_3_N_4_–MoS_2_) was amalgamated with a 3 mL of N-methyl-2-pyrrolidinone solution and coated on nickel foam substrates (1 cm × 1 cm, 110 ppi) that were purchased from the MTI Korea Group, Seoul, Republic of Korea. Next, each substrate (i.e., catalyst-coated nickel foam) was dried at 180 °C for 480 min. Moreover, a saturated calomel electrode (SCE) (i.e., reference electrode) and a coiled Pt wire (i.e., counter electrode) were also prepared to set up the three-electrode system. After preparing all the electrode setups, the electrocatalytic characteristics were examined using the electrochemical workstation. First, the electrochemical cyclic voltammetry (CV) characteristics of the synthesized catalysts were examined in a 0–0.5 V potential range, where the scan rate was also varied from 10 to 100 mV/s. Next, the linear sweep voltammetry (LSV) characteristics were assessed at a fixed scan rate of 1 mV/s within certain potential windows (i.e., OER: −0.1–1.2 V, HER: −1–−1.8 V). Additionally, the electrochemical impedance spectroscopy (EIS) tests were performed using an AC signal amplitude of 10 mV in the 1 Hz to 10 kHz frequency range in 1 M KOH. Furthermore, the chronopotentiometry measurements were conducted at different current densities (i.e., OER: 10–100 mA/cm^2^, HER: −10–−100 mA/cm^2^), for which each step was maintained for 10 min. Here, it should be noted that the reference was standardized to the reversible hydrogen electrode (RHE) scale. Furthermore, all the polarization values and their corresponding curves were *i_R_*-corrected. The electrochemical double-layer capacitance (*C_DL_*) and its corresponding electrochemically active surface area (*ECSA*) of the catalysts were determined from the non-Faradaic CV curves by using the following relationships [53,54,55]:

(1) $$J_{DL}=C_{DL}\times v/A$$

(2) $$ECSA=C_{DL}/C_{e}\text{,}$$

where *J_DL_*, *C_e_*, *v*, and *A* are the double-layer charging current, capacitance of the used electrolyte (0.04 mF/cm^2^ for KOH), the potential scan rate, and the area of electrodes, respectively. The overpotential (*η*) and the Tafel slope (*S_T_*) for the OER and HER were calculated using the following equations [14,56,57,58,59,60]:

(3) $$E_{RHE}=E_{SCE}+E_{SCE}^{0}+0.059pH$$

(4) $$\eta =E_{RHE}-1.23\;V\;\text{(for OER)}$$

(5) $$\eta =E_{RHE}\;\text{(for OER)}$$

(6) $$\eta =S_{T}\log\left(J\right)+c$$

where *E*^0^*_SCE_* is the SCE’s standard potential, *J* is the current density, *c* is the fitting parameter, and *E_RHE_* is the RHE’s standard potential.

## 3. Results and Discussion

The morphology and chemical composition of the bare 2D MoS_2_ nanosheets and 2D–2D gC_3_N_4_–MoS_2_ nanocomposites were characterized via FE-SEM and in situ EDX measurements. Figure 1b–e display the FE-SEM images of the pristine MoS_2_ nanosheets and the hybrid gC_3_N_4_–MoS_2_ nanocomposites. The MoS_2_ sample exhibited stacked-layer nanosheet-like structures with an average length of 200–400 nm (Figure 1b,c). After ultrasonicating the bare MoS_2_ nanosheets together with the gC_3_N_4_ nanosheets, the sample exhibited the interconnected and agglomerated structure of the 2D gC_3_N_4_-decorated 2D MoS_2_ nanocomposites (Figure 1d,e). Next, the elemental composition of the bare MoS_2_ nanosheets and gC_3_N_4_–MoS_2_ nanocomposites was investigated. As shown in the EDX profiles (Figure 1f,g), both samples revealed their own intrinsic Mo, S, N, and C constituents, demonstrating that the prepared materials were highly pure and free of other impurities. Here, it should be noted that Pt detected in both samples arose from the conductive coating for FE-SEM measurements, which was applied to avoid the electron charging effect.

**Figure 2:**
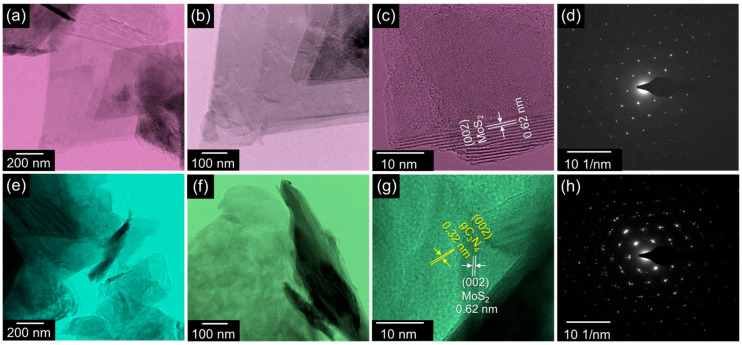
(**a**,**b**) Bright-field TEM images, (**c**) high-resolution TEM image, and (**d**) SAED pattern of MoS_2_. (**e**,**f**) Bright-field TEM image, (**g**) high-resolution TEM image, and (**h**) SAED pattern of gC_3_N_4_–MoS_2_.

The topography of both the bare 2D MoS_2_ nanosheets and 2D–2D gC_3_N_4_–MoS_2_ nanocomposites was further analyzed by TEM measurements. Figure 2a,b show the bright-field TEM images of the MoS_2_ nanosheets. The bare MoS_2_ exhibited a stacked-layer nanosheet morphology. In the high-resolution image (Figure 2c), the bare MoS_2_ shows an interlayer spacing of 0.62 nm, which corresponds to the (002) plane of hexagonal MoS_2_ [61]. The well-defined diffraction lattice indicates that the bare MoS_2_ nanosheets were single crystals corresponding to the hexagonal phase of MoS_2_ (Figure 2d). Unlike the bare MoS_2_ nanosheets, the hybrid gC_3_N_4_–MoS_2_ nanocomposites exhibited an interconnected and agglomerated structure of gC_3_N_4_ nanosheets decorated on stacked-layer MoS_2_ nanosheets (Figure 2e,f). From the high-resolution image of gC_3_N_4_–MoS_2_ (Figure 2g), the lattice spacings of MoS_2_ and gC_3_N_4_ were confirmed to be 0.62 and 0.32 nm, which are consistent with those of (002) MoS_2_ and (002) gC_3_N_4_, respectively. Moreover, the SAED pattern of gC_3_N_4_–MoS_2_ exhibited polycrystalline phases (Figure 2h) because of its microstructural hybridization.

To further understand the formation kinetics of the gC_3_N_4_–MoS_2_ nanocomposites, we discuss the chemical mechanisms implicated during the ultrasonication process. The sonochemical technique possesses several advantages, including rapid synthesis, low energy consumption, and the ability to generate highly dispersed nanostructures with uniform morphology. Ultrasonic irradiation induces acoustic cavitation, producing localized high temperatures and pressures that promote fast nucleation and prevent agglomeration. These features result in materials with high surface area, better crystallinity, and enhanced active site exposure—critical for improving electrocatalytic performance. In an aqueous solution, ultrasonication generates two significant radicals from water (i.e., hydrogen (H^*^) and hydroxyl (OH^*^) radicals). During the sonication of bulk materials, these H^*^ and OH^*^ radicals serve as reductants [62,63,64]. Consequently, bulk MoS_2_ (*n*MoS_2_) could be diminished into stacked-layer MoS_2_ nanosheets (MoS_2(n)_) under the high ultrasonic power in water. This sonochemical exfoliation process (i.e., micro-cavitation and shock waves) can be described by the following reactions [63,65,66,67,68]:

(7) $$H_{2}O\underset{Sonication}{\to }{OH}^{*}+H^{*}$$

(8) $$n{MoS}_{2}+{OH}^{*}+H^{*}\underset{Sonication}{\to }\;{MoS}_{2(n)}$$

(9) $$n{MoS}_{2}+{gC}_{3}N_{4}+{OH}^{*}+H^{*}\underset{Sonication}{\to }{MoS}_{2(n)}\text{–}{gC}_{3}N_{4}\text{–}{MoS}_{2(n)}\text{–}\cdots .$$

**Figure 3:**
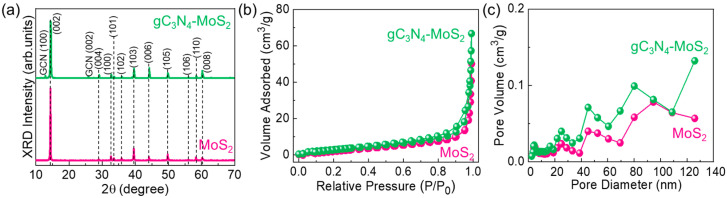
(**a**) XRD patterns, (**b**) N_2_-ADI characteristic curves, and (**c**) pore size distributions of MoS_2_ nanosheets and gC_3_N_4_–MoS_2_ nanocomposites.

Next, the crystallographic phases of pristine MoS_2_ nanosheets and gC_3_N_4_–MoS_2_ nanocomposites were investigated using powder XRD measurements. Figure 3a shows the XRD patterns of MoS_2_ and gC_3_N_4_–MoS_2_. Both samples revealed diffraction angles at 14.38°, 29.14°, 32.72°, 33.58°, 35.94°, 39.67°, 44.25°, 49.94°, 56.07°, 58.36°, and 60.23°, which are attributed to the (002), (004), (100), (101), (102), (103), (006), (105), (106), (110), and (008) planes of 2H-phase hexagonal MoS_2_, respectively (JCPDS No. 75-1539) [69,70,71]. The gC_3_N_4_–MoS_2_ nanocomposites displayed two additional diffraction peaks at 12.88° and 25.77°, attributed to the (100) and (002) planes of hexagonal gC_3_N_4_, respectively (JCPDS No. 87-1526) [72,73]. The XRD pattern of gC_3_N_4_–MoS_2_ displayed diffraction peaks from both MoS_2_ and gC_3_N_4_, confirming the successful fabrication of the gC_3_N_4_–MoS_2_ hybrid composite system. Moreover, no other secondary phases were observed in the fabricated materials, implying that the materials reflected high purity.

After confirming the successful hybridization of 2D gC_3_N_4_ and 2D MoS_2_, the textural characteristics were assessed through BET and BJH measurements using N_2_ adsorption–desorption isotherms (N_2_-ADIs). Figure 3b shows the N_2_-ADI curves of the bare MoS_2_ nanosheets and gC_3_N_4_–MoS_2_ nanocomposites. Both materials exhibited the Type-II isotherm characteristics, with a typical Type-H3 hysteresis loop (classified from IUPAC), demonstrating the distinctive features of mesoporous materials [74,75,76]. Based on the BET measurements, the specific surface areas of the bare MoS_2_ nanosheets and gC_3_N_4_–MoS_2_ nanocomposites were calculated to be 14 and 46 m^2^/g, respectively. Furthermore, BJH analysis revealed that the pore surface areas of the bare MoS_2_ nanosheets and gC_3_N_4_–MoS_2_ nanocomposites were 6 and 14 m^2^/g, respectively (Figure 3c). Additionally, the average pore diameter of MoS_2_ and gC_3_N_4_–MoS_2_ were 23.91 and 18.48 nm, respectively. Owing to the incorporation of layered gC_3_N_4_ nanosheets into the gC_3_N_4_–MoS_2_ composite system, the total pore volume was greater for gC_3_N_4_–MoS_2_ (0.09136 cm^3^/g) compared to that for MoS_2_ (0.06124 cm^3^/g). The unique porosity and high surface area of the gC_3_N_4_–MoS_2_ nanocomposites could be beneficial for enhancing their bifunctional water-splitting performance in alkaline media, which will be discussed later in detail.

**Figure 4:**
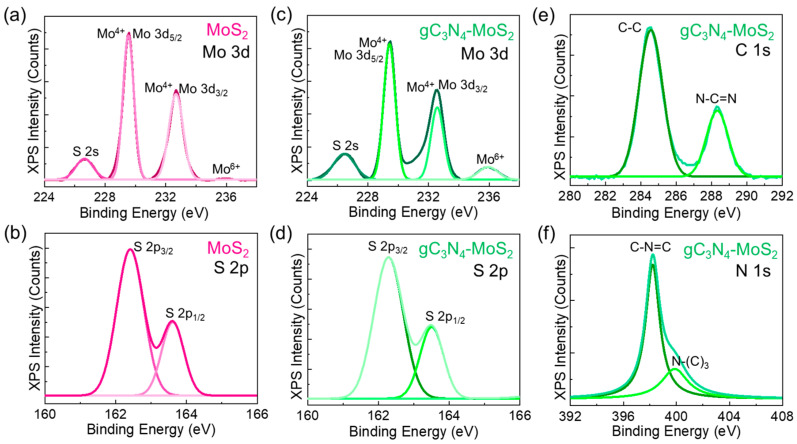
(**a**) Mo 3d and (**b**) S 2p core-level spectra of MoS_2_ nanosheets. (**c**) Mo 3d, (**d**) S 2p, (**e**) C 1s, and (**f**) N 1s core-level spectra of gC_3_N_4_–MoS_2_ nanocomposites.

The surface composition and ionic state interaction for both MoS_2_ and gC_3_N_4_–MoS_2_ were examined using XPS measurements. The XPS full survey spectrum of the bare MoS_2_ nanosheets and gC_3_N_4_–MoS_2_ nanocomposites clearly revealed their intrinsic elements, including Mo, S, C, N, and O (see Figure S1a,b). For the Mo 3d core-level spectra (Figure 4a), the bare MoS_2_ displayed two predominant peaks at 229.46 and 232.58 eV, associating with the Mo 3d_5/2_ and Mo 3d_3/2_ spin-orbit splitting states of Mo^4+^, respectively [77]. In addition, the small peak observed at 235.91 eV is correlated with the orbital electron of Mo^6+^, indicating the existence of small amounts of MoO_3_ due to the surface oxidation of MoS_2_ [78]. The S 2s peak observed at 226.64 eV corresponds to the hexagonal phase of MoS_2_ [79]. For S 2p (Figure 4b), the two peaks at 162.41 and 163.62 eV arise from the S 2p_3/2_ and S 2p_1/2_ orbit splitting states of the S^2−^ ion, respectively [80,81]. The hybrid gC_3_N_4_–MoS_2_ nanocomposites clearly revealed similar features of the Mo 3d (Figure 4c) and S 2p (Figure 4d) core-level spectra. In the case of C 1s (Figure 4e), gC_3_N_4_–MoS_2_ clearly exhibited two carbon peaks at 284.61 and 288.36 eV, attributed to the C–C and N–C=N bonds, respectively [41,82]. Moreover, as shown in Figure 4f, the N 1s core-level spectra contained two peaks at 398.24 and 399.93 eV, ascribed to the pyridinic C–N=C and triazine N–(C_3_) bonds, respectively [34,83]. Additionally, the oxygen-related peaks were observed at 532.15 and 533.92 eV, attributed to the Mo–O bond and chemisorbed oxygen [34,41], respectively (see Figure S1c,d). These results clearly indicate that the gC_3_N_4_ nanosheets are well-decorated on the MoS_2_ nanosheets in the composite system.

**Figure 5:**
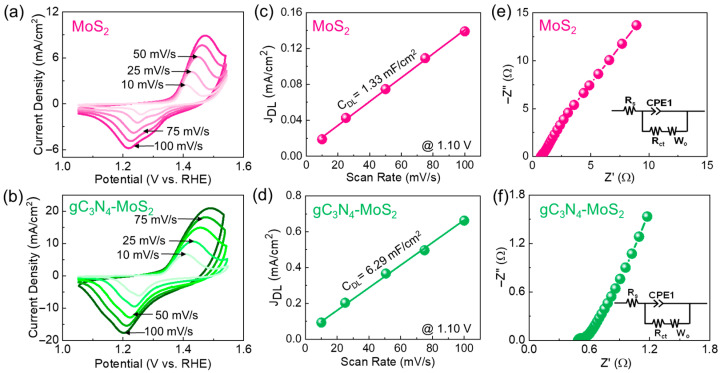
CV curves of (**a**) MoS_2_ and (**b**) gC_3_N_4_–MoS_2_. Non-faradaic *J_DL_* at 1.10 V as a function of the potential scan rate for (**c**) MoS_2_ and (**d**) gC_3_N_4_–MoS_2_. Nyquist plots of (**e**) MoS_2_ and (**f**) gC_3_N_4_–MoS_2_. The insets of (**e**,**f**) illustrate the equivalent circuits of the fabricated working electrodes.

To investigate the impact of 2D–2D gC_3_N_4_–MoS_2_ hybridization on its electrocatalytic performance, we assessed the electrochemical CV characteristics of the bare MoS_2_ nanosheets and gC_3_N_4_–MoS_2_ nanocomposites. As shown in Figure 5a,b, both the MoS_2_ and gC_3_N_4_–MoS_2_ catalysts display noticeable redox peaks in their CV curves. These peaks are mainly due to the electrochemical activity of the NF substrate, which undergoes a reversible Ni^2+^ to Ni^3+^ oxidation and Ni^3+^ to Ni^2+^ reduction in alkaline solution [84], while the catalysts may slightly influence the peak intensity or shape of the CV curves. These redox features are an indicative of the pseudocapacitive behavior that may affect both OER and HER activities. Here, it is noteworthy that the current density increased with an increasing scan rate. This means that the active catalyst material possesses a low diffusion resistance. Compared to the bare MoS_2_ catalyst, the gC_3_N_4_–MoS_2_ hybrid catalyst exhibited a wider CV window with a greater current response. This implies that the 2D–2D gC_3_N_4_–MoS_2_ catalyst possessed a higher number of active sites than did the MoS_2_ catalyst. We believe that the enhanced electrochemical activity of the 2D–2D gC_3_N_4_–MoS_2_ hybrid catalyst is due to two possible reasons: the increased number of active sites [67,85,86] and the increased electrical conductivity [14,35,36,60,87]. The former will be explained below, while the latter is discussed later in the EIS section.

To elucidate the improved electrocatalytic performance of the gC_3_N_4_–MoS_2_ hybrid catalyst, we first calculated the *ECSA* of the catalyst materials using Equations (1) and (2). The *ECSA* values were determined from the non-faradic CV regions (1.05 to 1.15 V) for both MoS_2_ and gC_3_N_4_–MoS_2_ (see Figure S2a,b). From the *J*_DL_ vs. *v* curves obtained from the non-faradaic CV region at approximately 1.10 V (Figure 5c,d), the *C_DL_* values were calculated to be 1.33 and 6.29 mF/cm^2^ for MoS_2_ and gC_3_N_4_–MoS_2_, respectively. Using Equations (1) and (2), the corresponding *ECSA* values of 33 and 158 cm^2^ were estimated for MoS_2_ and gC_3_N_4_–MoS_2_, respectively. This indicates that the gC_3_N_4_–MoS_2_ hybrid catalyst displayed a larger *ECSA* than did the bare MoS_2_ catalyst (see Figure S3). Hence, it can be conjectured that the hybridization of gC_3_N_4_ and MoS_2_ increased the number of electrochemically active sites in the entire composite medium of gC_3_N_4_–MoS_2_. In short, hybridizing MoS_2_ with gC_3_N_4_ enhanced the specific surface area compared to that of bare MoS_2_. gC_3_N_4_ helps prevent the natural tendency of MoS_2_ to restack by acting as a spacer, leading to a more open and porous structure [34,88]. This exposes more active edge sites, which are crucial for water splitting. The nitrogen-rich surface of gC_3_N_4_ also provides anchoring points for MoS_2_, ensuring strong interaction and uniform dispersion [89,90]. In addition, electronic coupling between the two facilitates efficient charge transfer across the interface, while the rough, crumpled morphology created during assembly further increases surface roughness and accessibility [91,92,93].

Next, the electrochemical resistive behavior of the fabricated catalysts was examined through the EIS measurements. As shown in Figure 5e,f, the Nyquist plots of both MoS_2_ and gC_3_N_4_–MoS_2_ revealed straight lines at the low-frequency region, while they exhibited no parabolic curves at the high-frequency region. The former is relevant to the charge-transfer characteristics, which are directly associated with the series resistance (*R*_s_) of the working electrode [34,94,95,96], and the latter is attributed to the electrolyte dispersion characteristics [36,67,97]. Through fitting the EIS data to the equivalent circuit model (Figure 5e,f, insets), the *R*_s_ values of MoS_2_ and gC_3_N_4_–MoS_2_ were estimated to be 0.71 and 0.48 Ω, respectively. Thus, it can be inferred that gC_3_N_4_–MoS_2_ possess a better charge-transfer characteristic than does MoS_2_. This can be interpreted as resulting from the large porosity and high electrical conductivity of the incorporated gC_3_N_4_ nanosheets.

**Figure 6:**
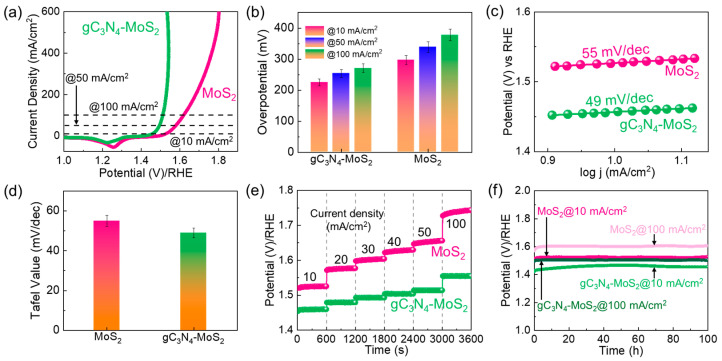
OER performance of fabricated electrocatalysts. (**a**) *i_R_*-corrected LSV curves, (**b**) overpotential comparison, (**c**) Tafel plots, (**d**) Tafel slope comparison, (**e**) chronopotentiometric profiles at 10–100 mA/cm^2^, and (**f**) long-term stability characteristics of MoS_2_ and gC_3_N_4_–MoS_2_.

The increased *ECSA* and the decreased *R*_s_ help to enhance rapid ion diffusion and swift electron transport, resulting in improved OER/HER performance. To verify this hypothesis, we performed LSV measurements for MoS_2_, gC_3_N_4_, and gC_3_N_4_–MoS_2_. Figure 6a shows the *i_R_*-corrected OER LSV curves of the bare MoS_2_ and gC_3_N_4_–MoS_2_ hybrid catalysts at 1 mV/s (see also Figure S4a for bare gC_3_N_4_). Using Equations (3) and (4), the *η*_10_ values of MoS_2_, gC_3_N_4_, and gC_3_N_4_–MoS_2_ were calculated to be 297, 325, and 225 mV, respectively, from the measured LSV curves at *J* = 10 mA/cm^2^. The gC_3_N_4_–MoS_2_ hybrid catalyst exhibited lower overpotential values (*η*_50_ = 254 mV and *η*_100_ = 271 mV), even at high current density (*J* = 50 and 100 mA/cm^2^), compared to those for MoS_2_ (*η*_50_ = 339 mV and *η*_100_ = 378 mV) and gC_3_N_4_ (*η*_50_ = 358 mV and *η*_100_ = 381 mV) (Figure 6b). Notably, the *η*_10_ values determined for MoS_2_ and gC_3_N_4_–MoS_2_ are in line with, and occasionally better than, established data from earlier studies (see Table S1). This means that the 2D–2D gC_3_N_4_–MoS_2_ catalyst exhibits outstanding intrinsic reaction kinetics, resulting in significant OER performance in alkaline electrolytes [98,99,100]. Improved OER performance can also be confirmed by determining the *S_T_* value. Using the Tafel equation in Equation (6), the small *S_T_* values of MoS_2_ (55 mV/dec), gC_3_N_4_ (58 mV/dec), and gC_3_N_4_–MoS_2_ (49 mV/dec) were determined from their Tafel curves (Figure 6c,d and Figure S4b). The achieved *S*_T_ values suggest that the MoS_2_, gC_3_N_4_, and gC_3_N_4_–MoS_2_ catalysts follow the combined Volmer–Heyrovsky mechanism. In particular, gC_3_N_4_–MoS_2_ exhibited a smaller *S*_T_ value than the values in the literature (Table S1). Namely, the 2D–2D gC_3_N_4_–MoS_2_ catalyst exhibited outstanding intrinsic reaction kinetics owing to its decreased charge-transfer resistance, large porosity, and increased active surface area. Compared to MoS_2_, the gC_3_N_4_–MoS_2_ catalyst exhibited lower *η* and smaller *S_T_* values, signifying the faster reaction rate of OH^−^ over the catalyst surface. The catalytic OER mechanism in an alkaline medium typically involves a four-electron process:

(10) $$M+{OH}^{-}\to M{OH}_{ads}+e^{-}$$

(11) $$M{OH}_{ads}+{OH}^{-}\to MO_{ads}+e^{-}+H_{2}O$$

(12) $$MO_{ads}+{OH}^{-}\to M{OOH}_{ads}+e^{-}$$

(13) $$M{OOH}_{ads}+{OH}^{-}\to M+e^{-}+O_{2}+H_{2}O$$

where M specifies an active site of the prepared catalysts, and M-OOH, M-O, and M-OH are the reaction intermediates on the catalysts’ surface.

The improved intrinsic reaction kinetics of the catalyst can also impact its chronopotentiometric characteristics. As shown in Figure 6e, the gC_3_N_4_–MoS_2_ catalyst displayed a smaller overpotential at each step (i.e., at different current densities) compared to that of the bare MoS_2_ catalyst. This proves that the incorporation of gC_3_N_4_ could aid in enhancing ion storage performance and catalytic activity. The long-term OER stability of the MoS_2_ and gC_3_N_4_–MoS_2_ catalysts was systematically evaluated through CP measurements conducted at 10 and 100 mA/cm^2^ for each 100 h, respectively. As shown in Figure 6f, both catalysts maintained stable performance and increased activity at diverse current densities. Furthermore, both samples exhibited almost indistinguishable LSV characteristic curves before and after the 100 h stability evaluation. (Figure S6). Compared to MoS_2_, however, gC_3_N_4_–MoS_2_ demonstrated superior long-term durability due to its trivial resistance and increased catalytic active areas. This indicates the excellent endurance of gC_3_N_4_–MoS_2_ in an alkaline medium.

**Figure 7:**
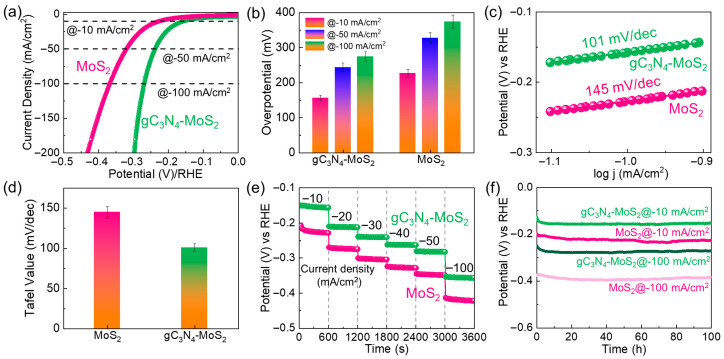
HER performance of fabricated electrocatalysts. (**a**) *i_R_*-corrected LSV curves, (**b**) overpotential comparison, (**c**) Tafel plots, (**d**) Tafel slope comparison, (**e**) chronopotentiometric profiles at −10–−100 mA/cm^2^, and (**f**) long-term stability characteristics of MoS_2_ and gC_3_N_4_–MoS_2_.

Next, we assessed the HER performance of the fabricated catalysts to examine their bifunctional water-splitting activity. Figure 7a shows the *i_R_*-corrected LSV curves of gC_3_N_4_ and gC_3_N_4_–MoS_2_ obtained during the HER process, which was conducted at a 1 mV/s scan rate in the KOH electrolyte (see also Figure S5a for bare gC_3_N_4_). Using Equations (3) and (5), the obtained *η*_10_ values of MoS_2_, gC_3_N_4_, and gC_3_N_4_–MoS_2_ were 228, 236, and 156 mV, respectively, at a current density of −10 mA/cm^2^. In addition, the gC_3_N_4_–MoS_2_ catalyst revealed smaller *η*_50_ (=244 mV) and *η*_100_ (=275 mV) values compared to those for the MoS_2_ catalyst (*η*_50_ = 327 mV and *η*_100_ = 374 mV) and gC_3_N_4_ catalyst (*η*_50_ = 321 mV and *η*_100_ = 378 mV) (Figure 7b). Furthermore, from the Tafel curves (Figure 7c,d and Figure S5b), the obtained *S*_T_ values for MoS_2_, gC_3_N_4_, and gC_3_N_4_–MoS_2_ were 145, 158, and 101 mV/dec, respectively. Notably, both *η* and *S*_T_ values obtained from gC_3_N_4_–MoS_2_ compare favorably with and, in certain cases, surpass those reported in prior literature. (Table S2). In both the chronopotentiometric HER test (Figure 7e) and the long-term HER stability test (Figure 7f and Figure S7), gC_3_N_4_–MoS_2_ also revealed better electrocatalytic activity than that of bare MoS_2_.

**Figure 8:**
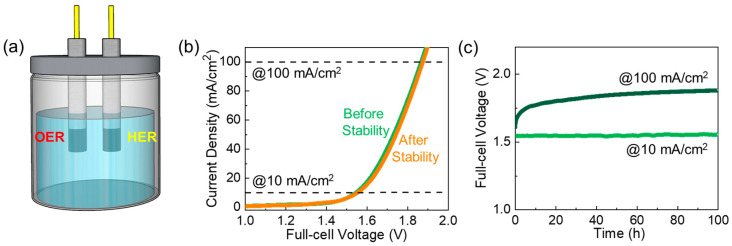
Overall water-splitting performance of gC_3_N_4_–MoS_2_||gC_3_N_4_–MoS_2_: (**a**) illustration of the two-electrode cell setup for OWS measurements, (**b**) LSV curves before and after stability testing, and (**c**) long-term stability characteristics over 100 h under current densities of 10 and 100 mA/cm^2^.

The outstanding bifunctional OER and HER performances of the 2D–2D gC_3_N_4_–MoS_2_ catalyst may allow for superior overall water-splitting performance. To verify this, we tested the overall water-splitting activities of gC_3_N_4_–MoS_2_ using a two-electrode configuration in 1M KOH (Figure 8a). Figure 8b displays the LSV curve of the gC_3_N_4_–MoS_2_|| gC_3_N_4_–MoS_2_ electrolyzer. Remarkably, the bifunctional gC_3_N_4_–MoS_2_||gC_3_N_4_–MoS_2_ electrolyzer could drive specific current densities with low full-cell voltage values (e.g., 10 mA/cm^2^ with 1.52 V and 100 mA/cm^2^ with 1.85 V). These results depict an adequate catalytic activity of gC_3_N_4_–MoS_2_ for efficient overall water electrolysis. The full-cell voltages achieved from the current gC_3_N_4_–MoS_2_||gC_3_N_4_–MoS_2_ electrolyzer are on par with or even lower than those previously reported for other metal oxide-based electrocatalysts (Table S3). Moreover, gC_3_N_4_–MoS_2_||gC_3_N_4_–MoS_2_ exhibited constant stability performance for 100 h at both 10 and 100 mA/cm^2^ (Figure 8c). After overall water electrolysis stability tests, the morphology of both MoS_2_ and gC_3_N_4_–MoS_2_ maintained its initial structure (see Figure S8a,b). In EDX analysis, it was confirmed that the intrinsic elements Mo, S, C, and N clearly appear, consistent with the composition of the synthesized catalysts. However, the detection of additional Ni, K, and O signals suggests interfacial interactions between the catalyst surface, the electrolyte, and the underlying Ni foam substrate during electrochemical testing (see Figure S8c,d). In XRD testing, the gC_3_N_4_–MoS_2_ still exhibited higher intensity compared to that of the bare MoS_2_, although both samples showed decreased XRD intensities after the completion of all the water electrolysis tests (see Figure S9a,b). The above results suggest that the sonochemically hybridized 2D–2D gC_3_N_4_–MoS_2_ nanocomposites exhibit excellent potential for use as a bifunctional electrocatalyst for superior overall water electrolysis.

## 4. Conclusions

High-performance 2D–2D gC_3_N_4_–MoS_2_ nanocomposites were successfully fabricated via a facile ultrasonication process using sonochemically synthesized MoS_2_ and pyrolytically derived gC_3_N_4_. The gC_3_N_4_–MoS_2_ nanocomposites exhibited excellent bifunctional OER and HER performance in 1 M KOH. Namely, the gC_3_N_4_–MoS_2_ catalyst demonstrated excellent OER performance (i.e., a low *η* of 225 mV and a small *S_T_* of 49 mV/dec), as well as outstanding HER performance (i.e., a low *η* of 156 mV and a small *S_T_* of 101 mV/dec). Moreover, the gC_3_N_4_–MoS_2_ electrocatalyst revealed the superb overall water splitting with a low full-cell voltage of 1.52 V at 10 mA/cm^2^. These results were attributed to both low *R*_s_ (0.48 Ω) and large *ECSA* (158 cm^2^), resulting from the hybridization of 2D MoS_2_ and highly conductive gC_3_N_4_. Additionally, gC_3_N_4_–MoS_2_ also demonstrated good long-term stability up to 100 h for overall water splitting. This work marks a significant improvement over previously reported MoS_2_-based catalysts by introducing a scalable, energy-efficient synthesis strategy that simultaneously improves catalytic activity and long-term durability. The adoption of a sonochemical approach offers a green, cost-effective, and industrially viable pathway for the fabrication of high-performance bifunctional electrocatalysts. Future research could explore compositional optimization, integration into membrane-based electrolyzer systems, and rigorous evaluation under industrially relevant conditions to facilitate the practical deployment of this catalyst in sustainable hydrogen production technologies.

## Data Availability

The original contributions presented in this study are included in the article/Supplementary Materials. Further inquiries can be directed to the corresponding authors.

## Supplementary Materials

The following supporting information can be downloaded at: https://www.mdpi.com/article/10.3390/ma18163775/s1, Figure S1: Full-survey XPS spectra of (a) MoS_2_ nanosheets and (b) gC_3_N_4_–MoS_2_ nanocomposites. O 1s core-level spectra of (c) MoS_2_ nanosheets and (d) gC_3_N_4_–MoS_2_ nanocomposites; Figure S2: Non-faradic CV curves of (a) MoS_2_ and (b) gC_3_N_4_–MoS_2_ catalysts; Figure S3: (a) i_R_-corrected OER LSV curve and (b) Tafel plot of the gC_3_N_4_ catalyst. Figure S4: (a) *i_R_*-corrected HER LSV curve and (b) Tafel plot of the gC_3_N_4_ catalyst. Figure S5: *ECSA* of the MoS_2_ and gC_3_N_4_–MoS_2_ catalysts; Figure S6: LSV curves of MoS_2_ and gC_3_N_4_–MoS_2_ before and after the OER stability test; Figure S7: LSV curves of MoS_2_ and gC_3_N_4_–MoS_2_ before and after the HER stability test; Figure S8: FE-SEM images of (a) MoS_2_ and (b) gC_3_N_4_–MoS_2_ after the stability test. EDX spectra of (c) MoS_2_ and (d) gC_3_N_4_–MoS_2_ after the stability test; Figure S9: XRD pattern of (a) MoS_2_ and (b) gC_3_N_4_–MoS_2_ after the stability test.; Table S1: Comparison of OER performance for MoS_2_, gC_3_N_4_, and gC_3_N_4_–MoS_2_ with that of previously reported electrocatalysts; Table S2: Comparison of HER performance for MoS_2_, gC_3_N_4_, and gC_3_N_4_–MoS_2_ with that of previously reported electrocatalysts; Table S3: Comparison of overall water splitting performance for MoS_2_, gC_3_N_4_, and gC_3_N_4_–MoS_2_ with that of previously reported electrocatalysts. References [101,102,103,104,105,106,107,108,109,110,111,112,113,114,115,116,117,118,119,120,121,122,123,124,125,126,127,128,129,130,131] have been cited in Supplementary file.
