# Supplementary material for: Highly Efficient Electrocatalyst of 2D–2D gC3N4–MoS2 Composites for Enhanced Overall Water Electrolysis
Source: Materials (Basel). 2025 Aug 12;18(16):3775. doi: 10.3390/ma18163775 (PMC12387503; doi:10.3390/ma18163775)
Supplement: Supplementary file 1 [file materials-18-03775-s001.zip › materials-3782648-supplementary.pdf]

# Supplementary Data

## Highly Efficient Electrocatalyst of 2D-2D $gC_3N_4$-$MoS_2$ Composites for Enhanced Overall Water Electrolysis

Sankar Sekar [1,2], Atsaya Shanmugam [1,2], Youngmin Lee [1,2*], Sejoon Lee[1,2*]

[1] *Division of System Semiconductor, Dongguk University-Seoul, Seoul 04620, Republic of Korea*

[2] *Quantum-functional Semiconductor Research Center, Dongguk University-Seoul, Seoul 04620, Republic of Korea*

*Corresponding Authors: ymlee@dongguk.edu (Y. Lee); sejoon@dongguk.edu (S. Lee)

■ **Surface Characteristics of $MoS_2$ and $gC_3N_4$-$MoS_2$**

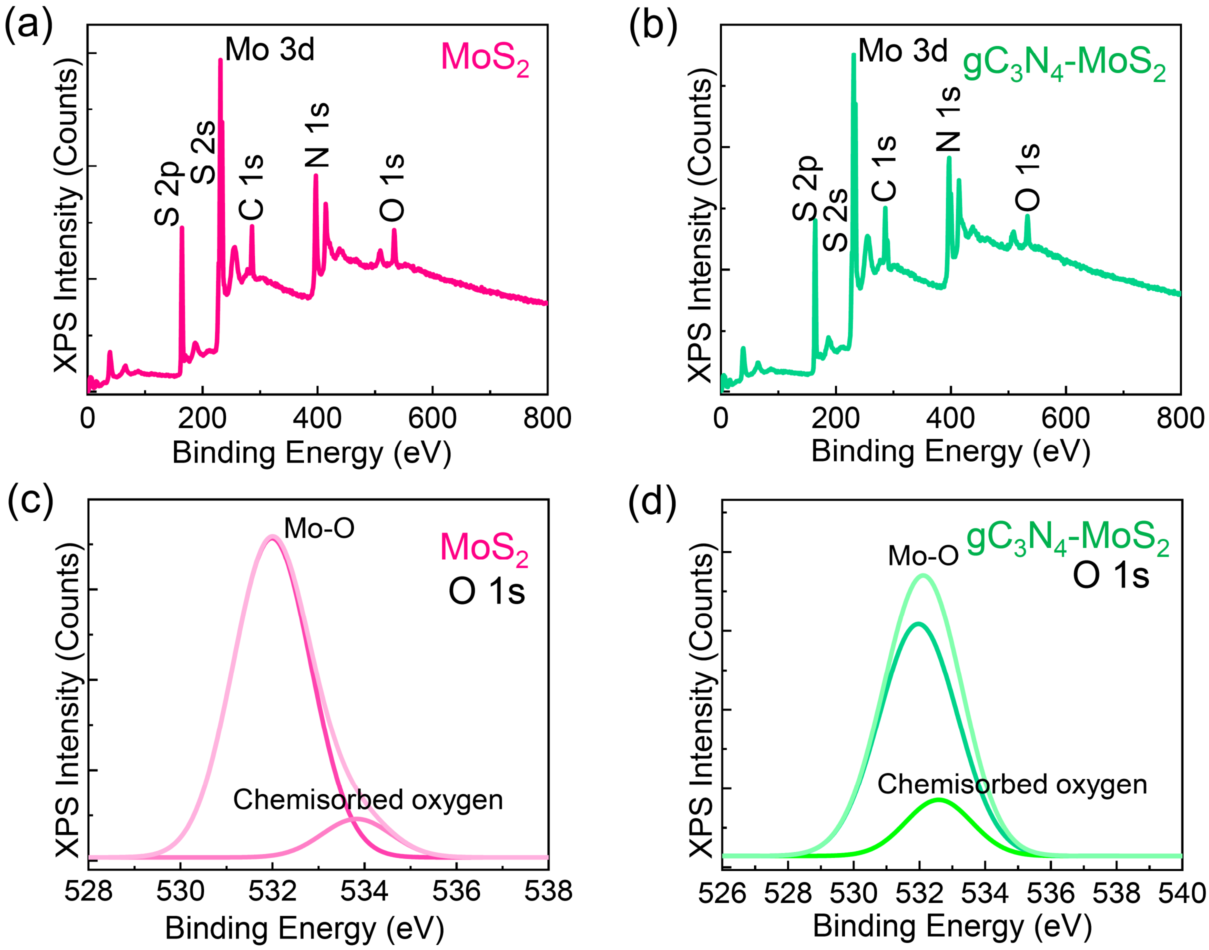


**Figure S1.** Full survey XPS spectra of (a) $MoS_2$ nanosheets and (b) $gC_3N_4$-$MoS_2$ nanocomposites. O 1s core levels spectra of (c) $MoS_2$ nanosheets and (d) $gC_3N_4$-$MoS_2$ nanocomposites.

■ **Electrochemical Properties of $MoS_2$ and $gC_3N_4$-$MoS_2$**

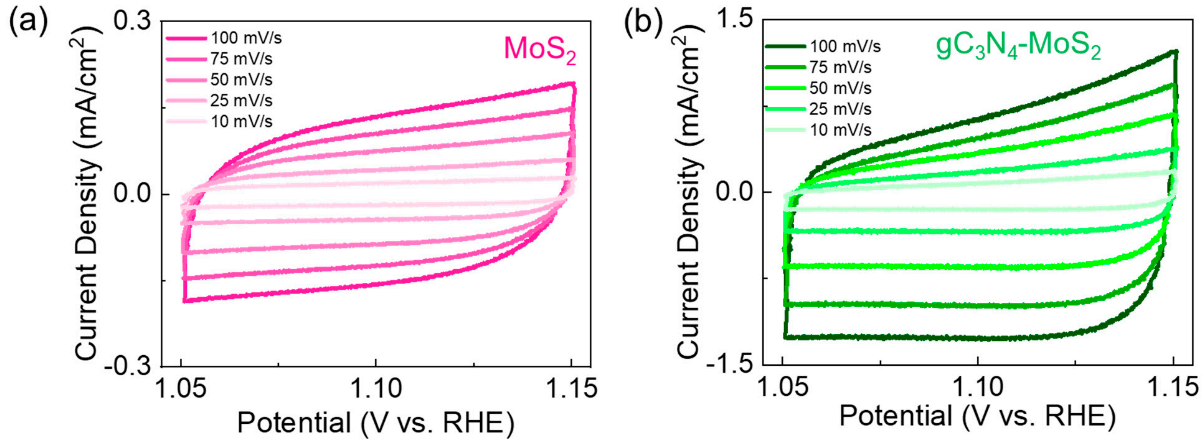


**Figure S2.** Non-faradic CV curves of (a) $MoS_2$ and (b) $gC_3N_4$-$MoS_2$ catalysts.

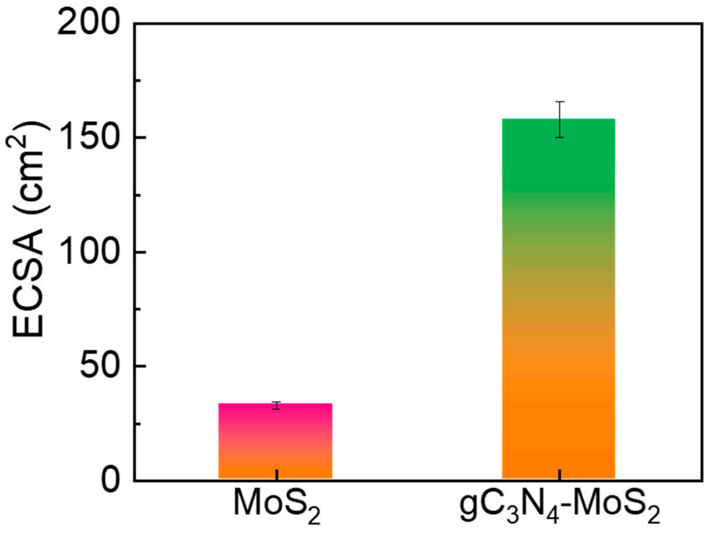


**Figure S3.** ECSA of the $MoS_2$ and $gC_3N_4$-$MoS_2$ catalysts.

■ **Electrocatalytic Properties of $gC_3N_4$**

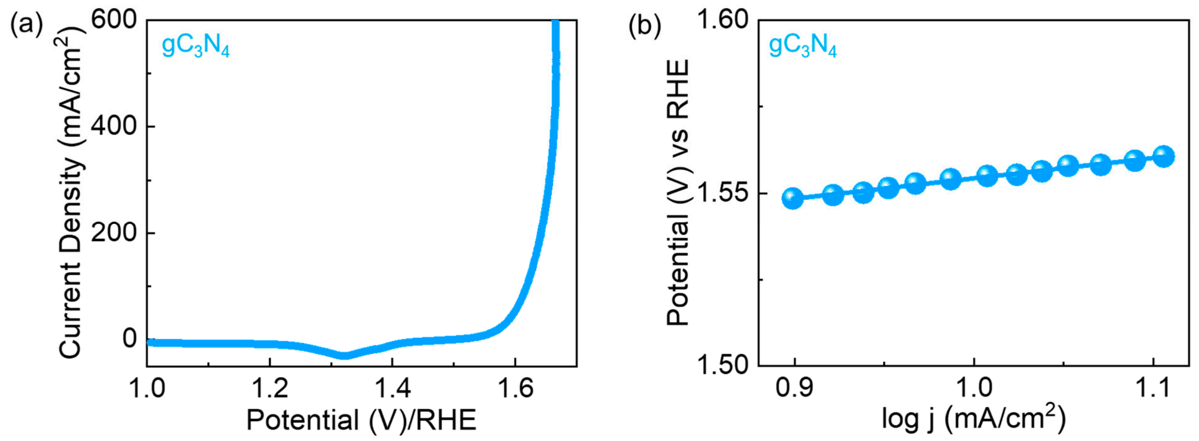


**Figure S4.** (a) iR-corrected OER LSV curve and (b) Tafel plot of the $gC_3N_4$ catalyst.

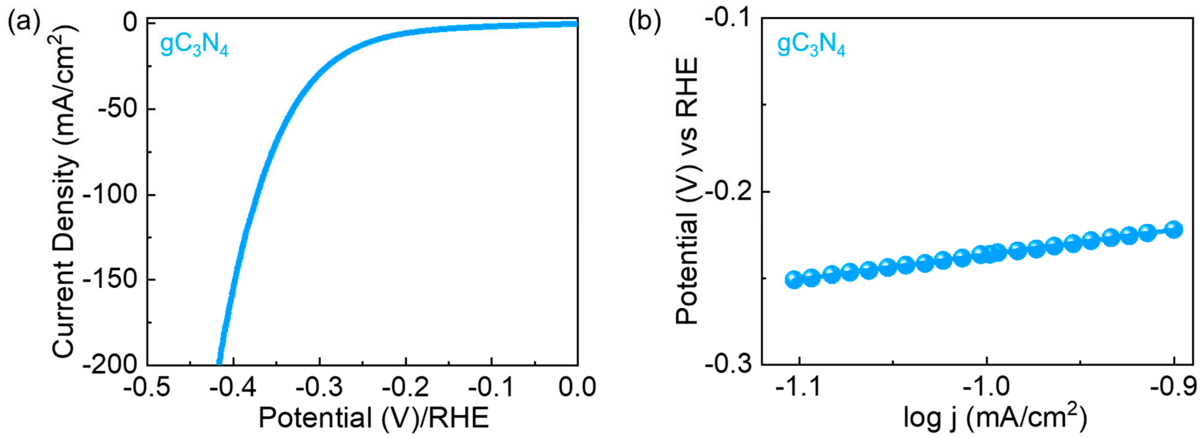


**Figure S5.** (a) iR-corrected HER LSV curve and (b) Tafel plot of the $gC_3N_4$ catalyst.

## ■ Electrocatalytic Properties of $MoS_2$ and $gC_3N_4$-$MoS_2$ before and after Stability Test

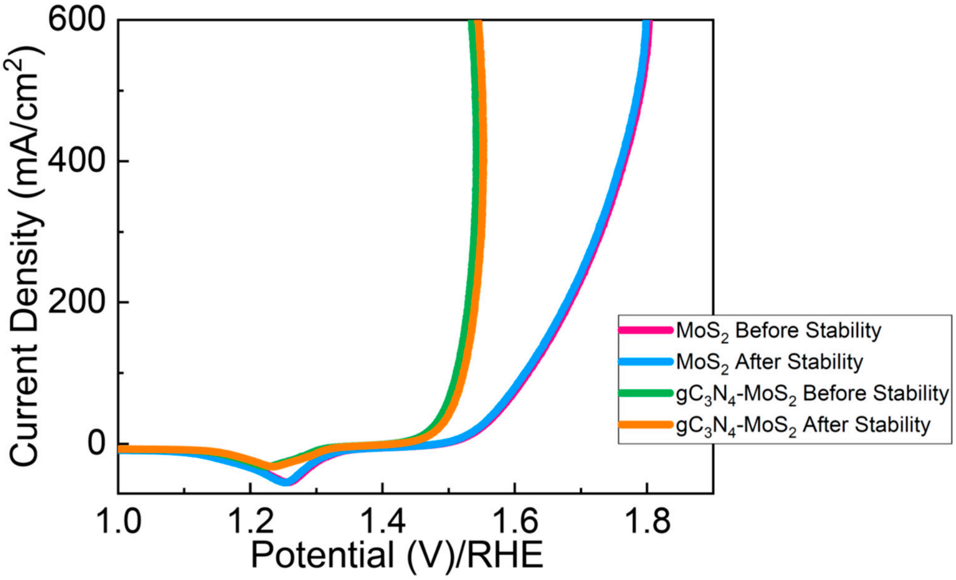


**Figure S6.** LSV curves of $MoS_2$ and $gC_3N_4$-$MoS_2$ before and after the OER stability test.

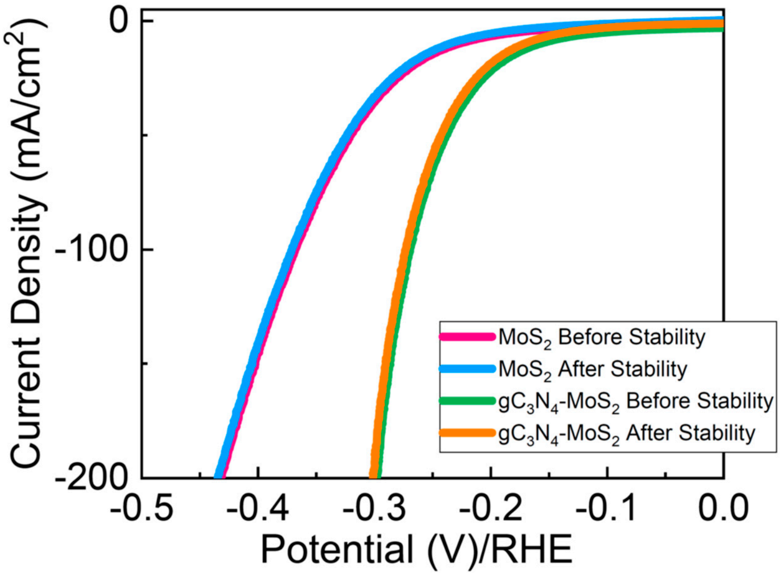


**Figure S7.** LSV curves of $MoS_2$ and $gC_3N_4$-$MoS_2$ before and after HER stability test.

■ **Morphological Properties of $MoS_2$ and $gC_3N_4$-$MoS_2$ after Stability Test**

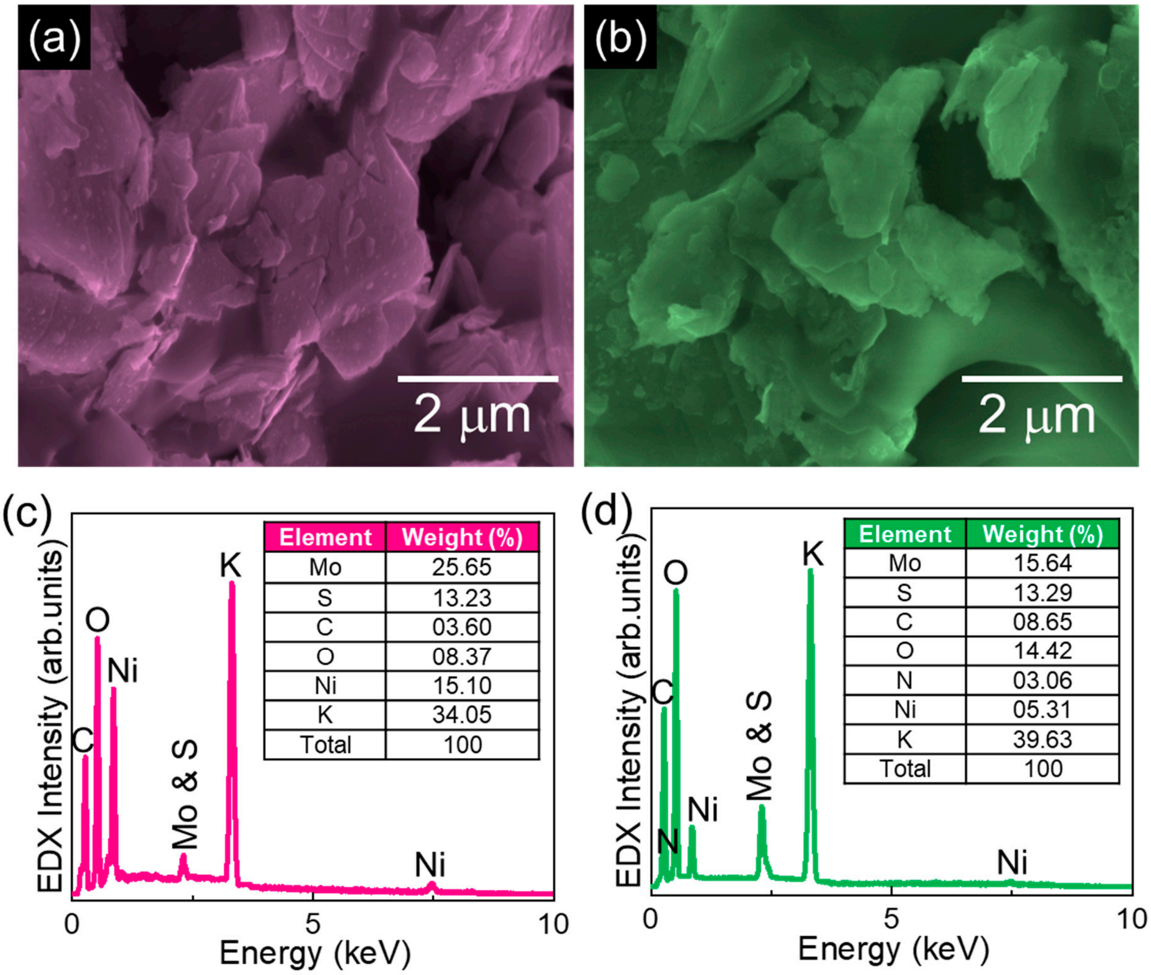


**Figure S8.** FE-SEM images of (a) $MoS_2$ and (b) $gC_3N_4$-$MoS_2$ after the stability test. EDX spectra of (c) $MoS_2$ and (d) $gC_3N_4$-$MoS_2$ after the stability test.

■ **Structural Properties of $MoS_2$ and $gC_3N_4$-$MoS_2$ after Stability Test**

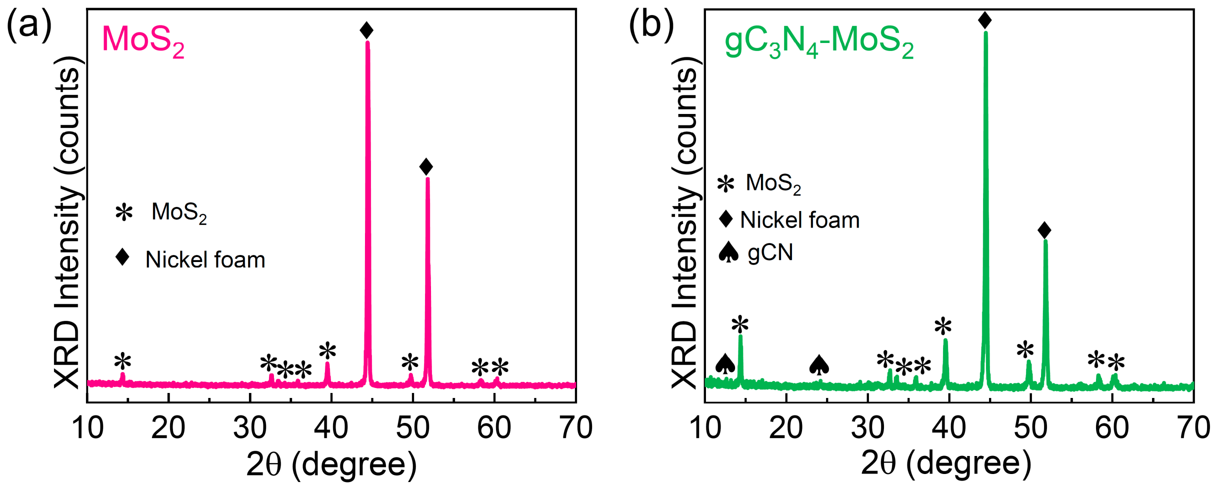


**Figure S9.** XRD pattern of (a) $MoS_2$ and (b) $gC_3N_4$-$MoS_2$ after the stability test.

## ■ Comparison of Electrocatalytic OER Activity for Various Materials

**Table S1.** Comparison of OER performance for $MoS_2$, $gC_3N_4$, and $gC_3N_4$-$MoS_2$ with previously reported electrocatalysts.

| Catalyst | Current density ($mA/cm^2$) | Overpotential $\eta_{10}$ (mV) | Tafel slope (mV/dec) | Electrolyte | Reference |
|---|---|---|---|---|---|
| **$gC_3N_4$-$MoS_2$** | **10** | **225** | **49** | **1 M KOH** | **This work** |
| **$MoS_2$** | **10** | **297** | **55** | **1 M KOH** | **This work** |
| **$gC_3N_4$** | **10** | **325** | **58** | **1 M KOH** | **This work** |
| $MoS_2$ Nano Islands | 10 | 300 | 45 | 1 M KOH | [96] |
| $MoS_2$@CoO | 10 | 325 | 129.9 | 1 M KOH | [101] |
| $MoO_2$-$Co_2Mo_3O_8$@C | 10 | 320 | 88 | 1 M KOH | [102] |
| AC-$V_2O_5$ | 10 | 230 | 54 | 1 M KOH | [60] |
| Fe-$MoS_2$ | 50 | 290 | 72 | 1 M KOH | [103] |
| Co-Ru-$MoS_2$ | 10 | 308 | 50 | 1 M KOH | [104] |
| AC-NiO | 10 | 320 | 49 | 1 M KOH | [67] |
| $MoO_3$/Ni-NiO | 100 | 347 | 96 | 1 M KOH | [105] |
| CF/VGSs/$MoS_2$/FeCoNi$(OH)_x$ | 500 | 225 | 29.2 | 1 M KOH | [106] |
| $MoS_2$/rGO | 10 | 120 | 171 | 1 M KOH | [36] |
| $CoS_x$@$Cu_2MoS_4$-$MoS_2$/NSG | 10 | 351.4 | 53.5 | 1 M KOH | [107] |
| $WO_3$/B-AC | 10 | 320 | 48 | 1 M KOH | [108] |
| CoSAs-$MoS_2$/TiN NRs | 10 | 340.6 | 81.2 | 1 M KOH | [109] |
| $MoS_2$ | 300 | 290 | 176 | 1 M KOH | [94] |
| Fe-($NiS_2$/$MoS_2$)/CNT | 10 | 234 | 60 | 1 M KOH | [110] |
| Co@$MoS_2$ | 10 | 270 | 41 | 1 M KOH | [111] |
| NiO/$gC_3N_4$ | 10 | 261 | 69.62 | 1 M KOH | [112] |
| Co-$gC_3N_4$ | 10 | 422.3 | 72.82 | 1 M KOH | [113] |

## ■ Comparison of Electrocatalytic HER Activity for Various Materials

**Table S2.** Comparison of HER performance for $MoS_2$, $gC_3N_4$, and $gC_3N_4$-$MoS_2$ with previously reported electrocatalysts.

| Catalyst | Current density ($mA/cm^2$) | Overpotential $\eta_{10}$ (mV) | Tafel slope (mV/dec) | Electrolyte | Reference |
|---|---|---|---|---|---|
| **$gC_3N_4$-$MoS_2$** | **10** | **156** | **101** | **1 M KOH** | **This work** |
| **$MoS_2$** | **10** | **228** | **145** | **1 M KOH** | **This work** |
| **$gC_3N_4$** | **10** | **236** | **158** | **1 M KOH** | **This work** |
| $MoS_2$/rGO | 10 | 242 | 59 | 1 M KOH | [36] |
| $WO_3$/B-AC | 10 | 360 | 14 | 1 M KOH | [108] |
| $MoS_2$ | 10 | 280 | 151 | 1 M KOH | [94] |
| $MoS_2$/g-$C_3N_4$ | 10 | 240 | 63 | 1 M KOH | [34] |
| $MoS_2$-$C_3N_4$ | 10 | 158 | 52 | 1 M KOH | [35] |
| g-$C_3N_4$/Ni(OH) | 10 | 341 | 131 | 1 M KOH | [45] |
| BiW@PEPS | 10 | 361 | 106 | 1 M KOH | [55] |
| $MoS_2$/$NiSe_2$/rGO | 10 | 127 | 73 | 1 M KOH | [114] |
| CoFe LDH-F | 10 | 255 | 40 | 1 M KOH | [115] |
| $CuBi_2O_4$ | 10 | 384 | 117 | 1 M KOH | [116] |
| FD-$MoS_2$ | 10 | 198 | 58.5 | 1 M KOH | [117] |
| $MoO_3$/AC | 10 | 353 | 124 | 1 M KOH | [98] |
| $MoS_{2-x}$/GCD-$MoS_{2-x}$ | 10 | 247 | 43 | 1 M KOH | [26] |
| S- $gC_3N_4$/NiV LDH | 10 | 560 | 79 | 1 M KOH | [118] |
| $MoS_2$/$WS_2$ NF | 10 | 251 | 61 | 1 M KOH | [119] |
| $NiMo_3S_4$ | 10 | 252 | 59 | 0.1 M KOH | [120] |
| $MoS_2$/MXene $Ti_3C_2$ | 10 | 207 | 28 | 1 M KOH | [121] |

## ■ Comparison of Overall Water Splitting Activity for Various Materials

**Table S3.** Comparison of OWS performance for $MoS_2$, $gC_3N_4$, and $gC_3N_4$-$MoS_2$ with previously reported electrocatalysts.

| Catalyst | Current density ($mA/cm^2$) | Electrolyte | Voltage (V) | Reference |
|---|---|---|---|---|
| **$gC_3N_4$-$MoS_2$** | **10** | **1 M KOH** | **1.52** | **This work** |
| $MoS_2/NiSe_2$/rGO | 10 | 1 M KOH | 1.52 | [114] |
| $MoS_2/NiFeS_2$ | 500 | 1 M KOH | 1.79 | [122] |
| $MoS_2/Cu_2O$ | 20 | 1 M KOH | 1.60 | [123] |
| $NiFeO_x(OH)_y$@$MoS_2$/rGO | 10 | 1 M KOH | 1.57 | [124] |
| $MoS_2/NiS_2$ | 10 | 1 M KOH | 1.56 | [125] |
| (Ni, Fe)$S_2$@$MoS_2$ | 10 | 1 M KOH | 1.56 | [126] |
| $Ni_2P$-$MoS_2$ HNSAs/CC | 10 | 1 M KOH | 1.57 | [127] |
| $MoS_2$@$Zn_{0.76}Co_{0.24}S$ | 10 | 1 M KOH | 1.45 | [128] |
| $Co_3O_4$@$MoS_2$/CC | 10 | 1 M KOH | 1.59 | [129] |
| N-$NiMoO_4/NiS_2$ | 10 | 1 M KOH | 1.60 | [130] |
| Ru-CoMOF@$MoS_2$ | 10 | 1 M KOH | 1.59 | [131] |
